# *Novo* plant-based mosquito repellent shows promise for exclusion of *Aedes* mosquitoes from “window” entry

**DOI:** 10.1093/jme/tjae137

**Published:** 2024-11-01

**Authors:** Sare I Yavasoglu, Martyn J Wood, James C Bull, Nergis Alkış, Emrecan Doğan, Abeer M Alkhaibari, Tariq M Butt

**Affiliations:** Department of Biology, Faculty of Science, Aydın Adnan Menderes University, 09010 Aydın, Türkiye; Institute of Molecular Biology and Biotechnology, Foundation for Research and Technology-Hellas, 73100 Heraklion, Greece; Department of Biosciences, Faculty of Science and Engineering, Swansea University, Singleton Park, Swansea SA2 8PP, UK; Department of Biology, Institute of Science, Aydın Adnan Menderes University, 09010 Aydın, Türkiye; Department of Biology, Institute of Science, Aydın Adnan Menderes University, 09010 Aydın, Türkiye; Department of Biology, Faculty of Science, University of Tabuk, Tabul 71491, Kingdom of Saudi Arabia; Department of Biosciences, Faculty of Science and Engineering, Swansea University, Singleton Park, Swansea SA2 8PP, UK

**Keywords:** *Aedes aegypti*, plant based, repellent olfactometric assay, “push-pull” strategy

## Abstract

Mosquitoes threaten over half of the world’s population through vectored diseases such as malaria, zika, yellow fever, dengue, and chikungunya. Mosquitoes have a highly developed olfactory system attuned to chemotaxis relating to host-seeking, mating, and oviposition behavior. In this study, we aimed to determine the spatial efficacy of 2 plant-based repellent blends (Blend3 and Blend4 that had previously been found to successfully repel Aedes, Anopheles and Culex mosquitoes in wind tunnel assays) in excluding *Aedes aegypti* from the window entry. A new cage system was developed for parallel “no-choice” and “choice” olfactometric assays. In the no-choice trial, Blends 3 and 4, as well as commercial products (N, N-diethyl-3-methylbenzamide, p-menthane-3,8-diol [PMD], 3-(N-n-butyl-N-acetyl)-amino-propionic acid ethyl ester, and 2-(2-hydroxyethyl)-1-methylpropylstyrene 1-piperidine carboxylate), were adsorbed into filter papers of different sizes and placed in a window created between 2 attached bug dorms. Then, the number of mosquitoes entering the window was counted through a 6-min period. In choice olfactometric assays, Blends 3, 4, and PMD were adsorbed into filter paper and the number of mosquitoes moving away from Blend 3 and PMD were compared. No-choice assays showed that Blend3 (*P* < 0.001) and Blend4 (*P* = 0.0012) were more repellent than the best commercial product PMD. Additionally, while Blend 4 was significantly more repellent than Blend 3 (*P* = 0.012) in the choice assay, overall, these 2 blends show promise as new repellents for the spatial exclusion of *Aedes aegypti* from window entry alone or as part of a “push-pull’’ strategy.

## Introduction

Mosquito-borne diseases (MBDs) pose a significant global challenge and require large investment from public health authorities. Over half the world’s population is at risk from MBDs such as malaria, dengue, chikungunya, yellow fever, Japanese encephalitis, and lymphatic filariasis (WHO 2023). Female mosquitoes generally require a blood meal to complete their reproductive cycle (Pitts et al. 2004), although there are certain exceptions where autogenous egg production is possible either in the first egg-laying cycle throughout all cycles (Tsuji et al. 1990, Ariani et al. 2015). Host location is largely mediated through olfactory cues, especially kairomonal compounds emanating from the human body such as carbon dioxide, ammonia, and a range of carboxylic acids including lactic acid (Dormont et al. 2021, Coutinho-Abreu et al. 2022, 2023). The behavioral responses of mosquitoes to components of human breath, sweat, and urine depend on the volatile organic compound (VOC) bouquet and specific concentrations of the VOCs therein (Qiu et al. 2011).

Effective mosquito control is a key component in preventing disease transmission. Traditional control strategies rely on the use of insecticide-treated nets and indoor residual spraying (Okumu and Moore 2011). Last decades, the development of insecticide resistance in mosquito populations and the negative effects of synthetic insecticides on the environment and human health (Senthil-Nathan 2020) have necessitated the development of novel ideas that can contribute to coherent integrated vector management (IVM) strategies. Beyond insecticidal control components, another key area of IVM relies on the use of personal protectants in the form of repellent compounds (Nalinya et al. 2022). Mosquitoes will enter houses via windows, doors, and eaves (Ogoma et al. 2010, Menger et al. 2016). Insect screens can provide a physical barrier, but their efficacy is highly dependent on mesh size; some screens are designed for the exclusion of larger insects such as house flies and therefore do not prevent the ingress of mosquitoes (Busvine 1965, Chanbang 2012, Dieke et al. 2022). Delicate, fine mesh screens and bed nets, designed to exclude small insects such as mosquitoes, sandflies, and midges can be easily damaged, offering potential entry routes for these pests (Sutcliffe et al. 2017, Dieke et al. 2022). Window screens too, a commonly used preventative mechanism, are often subject to external elements and degrade over time, providing further entry points. Incorporation of repellent products around key areas of damage in these systems could help in longer-term prevention of mosquito entry. Prior studies into such techniques produced encouraging results by using transfluthrin-treated fabric strips placed into the eaves of houses (Fillinger et al. 2023) or use of transfluthrin diffusers in military tents (Rajagopal et al. 2023). Unfortunately, transfluthrin is ineffective where mosquito populations have developed resistance to pyrethroids (McMillan et al. 2022).

Mosquito repellents can be formulated and deployed in more amenable forms such as gels, microencapsulated beads, polymer strings, or other dispensers (Mapossa et al. 2021) which could be easily clipped on or sprayed onto the mesh, bed net, or tent. The repellents could also be used as part of a “push-pull” strategy whereby mosquitoes are encouraged to enter through specific entry points where they could be intercepted and eliminated. The “push-pull” strategy has shown much promise in the management of crop pests (Khan et al. 2016), using repellents to drive a pest away from a host and redirecting it to an alternative that does not lead to disease or pest infestations (da Silva et al. 2022). Adaptation of this strategy within public health measures may well produce similar overall enhancements to IVM efficacy.

Many mosquito repellents based on synthetic chemicals including N, N-diethyl-3-methylbenzamide (DEET) and those developed from pyrethroid insecticides, impact on human health, pollute the environment, or become ineffective due to the development of resistance (Deletre et al. 2019, Black et al. 2021, Gan et al. 2021, Kim et al. 2023). While the development of pyrethroid resistance has generally not been understood to affect repellent actions (Bowman et al. 2018), some cases of altered behavioral response have been reported (Deletre et al. 2019). This has prompted an interest in more benign alternatives, with much attention being focused on botanicals, including essential oils and plant-derived compounds such as geraniol and citronella (Müller et al. 2009, Pohlit et al. 2011, Chellappandian et al. 2018, Mishra et al. 2023). Many plant-based compounds used as repellents are also used in the perfume industry or are active ingredients of air fresheners and soaps (Sarkic and Stappen 2018, Sharmeen et al. 2021). Mosquito repellents with attractive properties in terms of human olfaction are likely to be more acceptable to consumers and the general public and, therefore, more likely to be deployed in urban environments or as personal protectants. Recently, Wood et al. (2024) developed 2 blends (Blends 3 and 4) of natural compounds from spruce (*Picea sitchensis*) which repelled host-seeking *Aedes, Culex* and *Anopheles* mosquitoes on contact, but also displayed weak spatial repellency in secondary assays.

The current study focusses on evaluation of these blends, developed in Wood et al. (2024), using a more robust spatial-assay to determine their potential in preventing entry of mosquitoes through windows or other entry points in the house. The study establishes what percentage area of a window needs to be treated to prevent mosquitoes from entering despite the presence of a human host. Generating a full understanding of the mechanics of repellent interactions, and how interspacing may affect the efficacy of spatially repellent products, is a vital step in the development of optimized dispenser and application technologies and protocols. It is hoped that the information generated in the following studies can be used to enhance development and deployment of next generation naturally derived repellents in both personal protective strategies and wider public health measures such as “push-pull” control.

## Materials and Methods

### Mosquitoes

*Aedes aegypti* (Strain: Bora Bora) mosquitoes were obtained from Aydın Adnan Menderes University. Larvae were fed with ground fish food (Tetramin, Germany) and raised as a distinct colony in a controlled temperature room at 27 ± 2 °C, 80 ± 10% Relative Humidity (RH) and a 12:12 Light:Dark (LD) photoperiod. A 10% sucrose solution was provided for mosquito sustenance throughout their adult life stages.

All mosquitoes used in experiments were recently mated, 5–7 days post emergence, and had never been provided a blood meal. Adult females were selected for sensitivity prior to assay. This was achieved through a human volunteer placing their arms near to the edge of the cage, and those mosquitoes that responded with landing-probing behavior within 30 s were removed to a separate enclosure and starved for 12 h before the onset of assay.

### Spruce Derived Blends 3 and 4 and Commercial Repellents

Chemicals required to create Blends 3 and 4 for the assay were purchased from Sigma Aldrich (Merck, Germany). Blends 3 and 4 were prepared as outlined in Wood et al. (2024). Briefly, Blend 3 (Borneol [CAS number: 464-43-7], Bornyl Acetate [CAS number: 5655-61-8], Eugenol [CAS number: 97-53-0], Isoeugenol [CAS number: 97-54-1]) and Blend 4 (Borneol, Bornyl Acetate, Eugenol, Isoeugenol, Camphor [CAS number: 76-22-2]) were prepared as racemic mixtures of each constituent ingredient in pyrex glass flasks. Prior to assay, blends were diluted in a 1:1 ratio with absolute ethanol (HPLC grade, Sigma Aldrich, Merck, Germany).

The commercial repellents p-menthane-3,8-diol (PMD) (Incognito, Holland & Barrett, UK), 2-(2-hydroxyethyl)-1-methylpropylstyrene 1-piperidine carboxylate (Picaridin) (Autan multi insect repellent, 20%, SC Johnson, UK), 3-(N-n-butyl-N-acetyl)-amino-propionic acid ethyl ester (IR3535) (Jungle Formula, 20%, Omega Pharma, UK), and DEET (OFF sportsmen deep woods, 30%, SC Johnson, UK) were selected as positive controls.

### No-Choice Olfactometric Assay

No-choice assays were designed to assess mosquito approach and landing behaviors when variations in repellent coverage were applied to a “window” through which mosquitoes were able to gain entry to a separate port in response to human odors passing through the whole experimental arena.

**Fig. 1.**
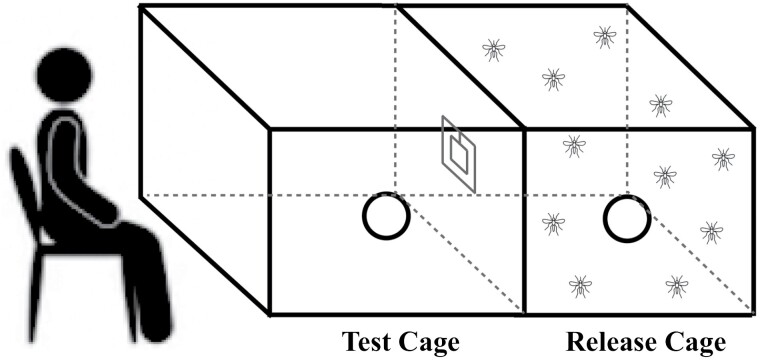
Schematic diagram of no-choice olfactometric assay.

Two 50 × 50 × 50 cm bug dorms (Watkins & Doncaster, UK) were securely attached along 1 edge, and a 10 × 10 cm “window” cut between the 2 bug dorms (Fig. 1). Varied proportions of the window were blocked by filter paper (4 × 10 cm, 5 × 10 cm, 6 × 10 cm) containing 1 of the repellents. Total coverage of the window area with repellent-infused filter paper equated to 40%, 50%, or 60% of the total window area. Treated filter papers were inserted in the center of the window; with the long side placed horizontally and the short side vertically. In total, 100 µl/9 cm^−2^ dosage of each repellent was applied on each filter paper. These ratios were 444.4 µl for 40 cm^2^ filter paper, 555.5 µl for 50 cm^2^ filter paper, and 666.6 µl for 60 cm^2^ filter paper. Positive controls included PMD, DEET, IR3535, and Picaridin. Since Blends 3 and 4 were prepared with ethanol, negative controls consisted of filter paper treated with pure ethanol in equal proportions to those used in Blend 3 and Blend 4 assays. Seventy-five mosquitoes were released into the release chamber of the experimental arena, with 1 of 3 human volunteers sitting at the “test” side of the arena. Volunteers did not have a shower, wear any scents, or be exposed to tobacco smoke for 12 h prior to the start of assay. Orientation of the set up and the volunteers were rotated at random between each replicate of each trial. Mosquitoes entering the “test” area via the window were counted, with total “catch” taken every minute for a total of 6 min. Five replicate experiments were conducted for each treatment included within the experiment. Between each experiments mosquitoes were left for 20 min for ventilation.

**Fig. 2.**
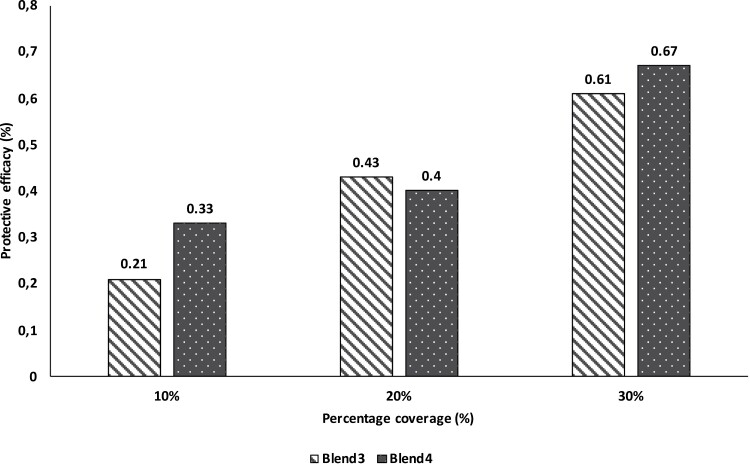
Protective efficacy of Blend 3 and Blend 4 with 10%, 20%, and 30% window coverage in 3 min.

Prior to the no-choice assay, the efficacy of 10%, 20%, and 30% coverage of filter paper with blends 3 and 4 was tested to determine coarse optimal efficacy for the full experiment thereafter (Fig. 2). In order to determine the role of effects of the filter paper as a potential physical barrier, the attractiveness of volunteers was also measured with 40%, 50%, and 60% window coverage alongside another assay where no filter paper was included, were tested with a total of 5 replicate studies (Supplementary Tables 1, 2, 3, and 4).

Mosquitoes attracted to the volunteers in the presence of repellent on the filter paper (N_T_) and in the absence of repellent on the filter paper (control) (N_C_) were counted and recorded. Protection efficacy (PE) was calculated as PE = 1—N_T_/N_C_.

### Choice Olfactometric Assay

**Fig. 3.**
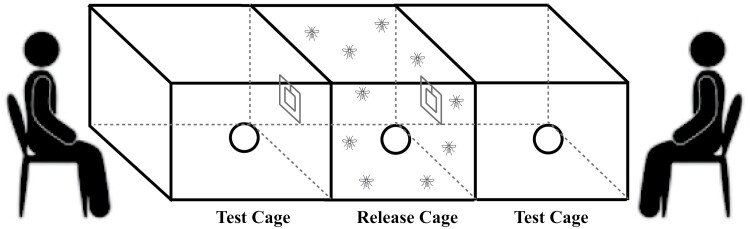
Schematic diagram of choice olfactometric assay.

A secondary set of experiments was conducted to determine the behavior of the mosquitoes when given a choice between 2 volunteers at either end of a 3-cage setup—described hereafter as the “choice” assay. Three cages were setup attached to each other lengthways as in the first set of experiments, excepting that the central cage, the “release” cage, had a 10 × 10 cm window cut on opposing sides to allow mosquitoes to make a “choice” in their direction of flight (Fig. 3). Seventy-five unfed adult female *Ae. aegypti* were released in the central chamber at the start of the assay.

The most effective commercial repellent from the first set of experiments, PMD, was used as the positive control repellent against treatments of either Blend 3 or Blend 4. Firstly, the experimental set was conducted as per the initial set of “no-choice” experiments, with 40% coverage of the window with the filter papers and repellents. Thereafter, however, experiments were continued with 30% coverage, based on the idea that strongly effective results were obtained with 40% coverage, and a drop to 30% should provide effective responses, enhance sensitivity and reduce chemical input. Secondary experimental sets were conducted in the same manner—with 30% coverage of the window with filter papers containing repellents—but with *novo* blends assayed against PMD in a single experiment using 2 volunteers, 1 at each end of the experimental arena and with 2 airflows directed from behind each volunteer. The attraction level of the volunteers’ and side effect were also tested prior to the assay to prevent any bias between right and left side of the test system (Supplementary Tables 3, 4, 5 and 6).

Six replicate experiments were conducted, and additional negative controls were included whereby Blend 3, Blend 4, and PMD were tested without a repellent counterpart, only a filter paper soaked with the diluent ethanol. Results were taken after a 3-min exposure period.

Mosquitoes attracted to the volunteers were counted and recorded. Number of mosquitoes attracted by any side of the test system was calculated as % mosquito number.

### Statistical Analysis

An attractiveness of volunteers, effect of side, and efficacy of filter papers against the window without filter paper were tested through paired samples t-tests based on the results obtained by Shapiro–Wilk test and Kolmogrov–Smirnov test.

In the no-choice olfactometric assay, the effects of treatment, window coverage, and time since the start of the assay were tested on mosquito attraction count data (N_T_ and N_C_). This was preferable to analyzing their effects on calculated PE, since PE was based on mean counts across replicates (reducing statistical power) and to prevent statistical challenges stemming from potential cases where N_T_ > N_C_, resulting in PE < 0 (even though this was not observed, null distributions used as the basis of statistical modeling would require the assumption that 0 < PE < 1).

Analysis was performed using negative binomial Generalised Linear Mixed-Effects Models (GLMM’s). The response variable was mosquito counts. Treatment and window coverage were fitted as categorical fixed effects. Time was fitted as a continuous fixed effect. Control *vs*. treatment was also included as a categorical fixed effect. All fixed effects were fitted as main effects and fully interacting. Controls and treatments were grouped as treatment blocks (i.e., each treatment was assessed against its own set of control replicates), fitted as a random effect. Temporal autocorrelation was also modeled as a first-order autoregressive process (AR1). Fixed effects fitted in the initial, full model were retained or dropped, based on Akaike Information Criterion corrected for small samples sizes, AICc (Mazerolle 2023). Here, this also matched a p-value based assessment of terms. Pairwise comparisons between factor levels of categorical variables in the final model were assessed using Tukey familywise error rate adjustment. GLMM’s were developed using the glmmTMB package (Brooks et al. 2017) and model comparisons made using the AICcmodavg package (Mazerolle 2023) in R version 4.4.0 (R Core Team 2024).

Following statistical analysis of N_T_ and N_C_, we calculated PE based on count model predictions, where PE = 1—N_T_/N_C_.

The choice assay was assessed using multinomial regression, modeling, and contrasting differences in the probability of individual mosquitoes being observed in each of the 3 linked cages (2 test cages and the release cage). This was performed using the mlogit package (Croissant 2020) in R, with individuals grouped by experimental replicate.

## Results

### Preliminary Analysis of Experimental Setup

In total, 47.06, 48.3%, and 48.8% of the 75 female mosquitoes passed through the window with 40, 50, and 60% filter paper coverage respectively; while 52.52% mosquitoes passed through the window containing no filter paper in the no-choice experiments. There was no significant difference between responses in assays either “with” or “without” filter papers inserted into the window (Supplementary Tables 1 and 2). The response was rapid (< 3 min) with the mosquitoes easily navigating through the “window” between the release and test cages.

In follow on choice assays, 37.8% and 37.1% of mosquitoes were attracted by volunteers 1 and 2, respectively. There were no significant differences between the attractiveness levels of each volunteer (t_df=7_ = 0.164, *P* = 0.875) (Supplementary Tables 3 and 4). Results also showed 36.8% and 38.2% of mosquitoes were attracted by right and left arms of the cages, respectively which was showing no bias between right and left arms of the test system (*t*_df=7_ = 0.483, *P* = 0.644) (Supplementary Tables 5 and 6).

### No-Choice Olfactometric Assay

**Fig. 4.**
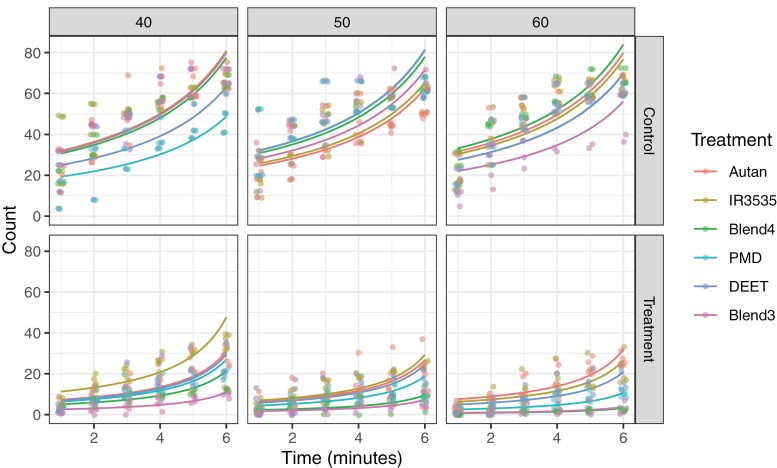
No-choice olfactometric assay mosquito counts over time for control (N_C_) and treatment (N_T_) cages (top row of panels *vs*. bottom row), for 40%, 50%, and 60% window coverage (left, middle, and right panels). Count data are displayed with a small amount of horizontal “jitter” to reveal points occupying the same location. Solid lines represent GLMM predictions.

In control cages, mosquito counts (N_C_) increased over time since the start of the assay, as expected, but varied randomly between sets of controls in each of the treatment blocks and there was no obvious effect of window coverage, indicating no unintended biases between blocks of controls (Fig. 4, see the ordering of the different coloured treatments across left, middle, and right panels on the top row). By comparison, in treatment cages, mosquito counts (N_T_) also increased over time but less steeply than with N_C_ for all treatments (Fig. 4, top row of panels *vs*. bottom row; “control *vs*. treatment”: time interaction, χ^2^_df = 1_ = 80.9, *P* < 0.001) and showed increasing repellence (lower N_T_) with increasing coverage (Fig. 4, “40” *vs*. “50” *vs*. “60” panels, bottom row; “control *vs*. treatment”: treatment: coverage interaction, χ^2^_df=10_ = 126.3, *P* < 0.001). Importantly, N_T_ was significantly lower with Blends 3 and 4 than commercial repellents (Pairwise comparisons in Supplementary Information). Unlike commercial repellents, Blends 3 and 4 remained close to 100% repellent throughout the length of the assay in cases of 60% window coverage (pink and green lines in the bottom-right panel of Fig. 4). GLMM analysis of deviance table is given in Supplementary Table 7.

**Fig. 5.**
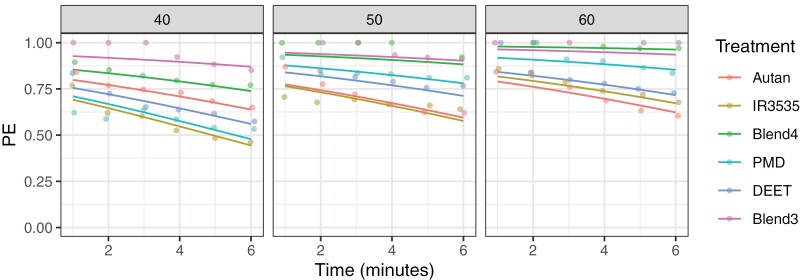
PE are shown as solid lines, based on mosquito count data GLMM predictions for 40%, 50%, and 60% window coverage (left, middle, and right panels). Data points show PE based on averaging observed N_T_ and N_C_ counts across 5 replicate assays for each experimental combination of treatment and window coverage and are displayed with a small amount of horizontal “jitter” to reveal points occupying the same location.

PE was calculated, based on count (N_T_*vs*. N_C_) model predictions, and shown in Fig. 5. While all products displayed repellence in our assays, Blends 3 and 4 clearly out-performed commercial repellents throughout the assay period, irrespective of window coverage level. Blend 4 (Fig. 5, green line across left, middle, and right panels) showed greater improvement in efficacy with increasing window coverage than Blend 3 (Fig. 5, pink lines) but the 2 blends’ efficacies were virtually indistinguishable at 50% and 60% coverage, remaining close to or better than 90% repellent without substantial drop-off through the length of the assay: By day 6, PE (95% c.i.) for Blend 3 = 0.90 (0.87, 0.93) and for Blend 4 = 0.88 (0.85, 0.91) at 50% coverage, and PE (95% c.i.) for Blend 3 = 0.94 (0.91, 0.96) and for Blend 4 = 0.96 (0.95, 0.97) at 60% coverage.

### Choice Olfactometric Assay

**Fig. 6.**
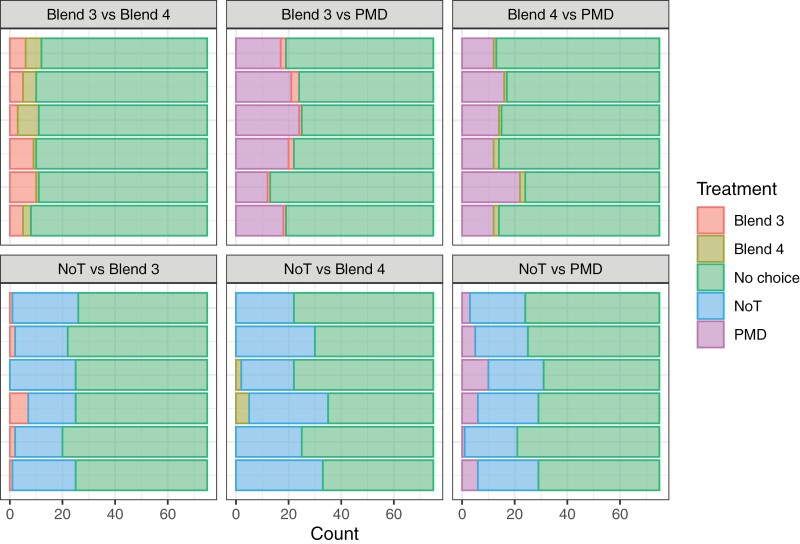
Choice olfactometric assay. Pairs of repellent (Blend 3, Blend 4, and PMD or no treatment, NoT) were compared using a 3-chamber setup. The number of mosquitoes (total = 75) observed in each chamber are shaded, with the far right-hand section of each sub-figure representing individuals observed in the release cage at the end of the trial (”no choice”). Six replicate trials for each treatment pair are shown up the y-axis.

All 3 treatments assessed (Blend 3, Blend 4, and PMD) were significantly more repellent than no treatment (NoT): Blend 3 *vs*. NoT, odds ratio = 0.149, z = −3.02, *P* = 0.003; Blend 4 *vs*. NoT, odds ratio = 0.047, z = −3.64, *P* < 0.001; PMD *vs*. NoT, odds ratio = 0.225, z = −3.27, *P* = 0.001 (Fig. 6, bottom row of panels).

Both Blend 3 (odds ratio = 0.053, z = -3.43, *P* < 0.001) and Blend 4 (odds ratio = 0.162, z = −2.50, *P* = 0.012) were significantly more repellent than PMD. Finally, Blend 4 was more repellent than Blend 3 when directly compared (odds ratio = 0.186, z = −2.50, *P* = 0.012) (Fig. 6, top row of panels).

## Discussion

Human-baited mosquito repellent assays are superior to assays using artificial lures, independent of assay design (Okumu et al. 2009). Mosquitoes are lured into houses by human odors, usually entering through open windows, doors, or eaves in the roof (Njie et al. 2009, Wanzirah et al. 2015). Insect screens placed in front of windows or doors can play an important role in excluding mosquitoes (Barreaux et al. 2020). The efficacy of insect screens, and bed nets, depends on the mesh size and if the mesh is treated with a pesticide or repellent. For example, Hossain and Curtis (1989) found that permethrin impregnated nylon netting of 4 mm or 6 mm mesh size had a strong toxic and excito-repellent effect on *Anopheles gambiae* but the effects were much weaker when the mesh size was 13 mm. The current study showed that blends 3 and 4, based on spruce-derived compounds, significantly reduced hungry *Aedes* females from reaching a human volunteer, but the degree of exclusion depended on the area treated. Blends 3 and 4 were more efficacious than commercial repellents but similar results were obtained at the 60% coverage, especially with PMD which is supposed to have some spatial repellent properties (Menger et al. 2014). However, the choice studies revealed that Blends 3 and 4 were significantly better than PMD as spatial repellents (Fig. 6). This confirms earlier assays that these blends may have weak spatial repellent properties, but that these are nevertheless superior to current commercial products (Wood et al. 2024).

The failure to exclude all mosquitoes is not unique to this study. This has been observed with many other repellents independent of assay design. For example, a 2.5% catnip and homopiperazine mix significantly decreased the ability of mosquitoes to find host odors emanating from a human finger by up to 96.7% (Obermayr et al. 2012). This repellence is similar to that obtained with Blends 3 and 4 using human volunteers.

Since a few mosquitoes did cross this physio-chemical barrier illustrates the tenacity of mosquitoes to locate a blood meal. The no-choice study gave some indication as to the area that needed to be treated to exclude mosquitoes from entry through windows or course mesh screens. Dispenser designs hinge on the behavioral specifics of the target species or group, information relating the spatial efficacy of repellent compounds provided herein can contribute to the development of polymers or reservoir technologies that can be effectively spaced and integrated in windows, screens, or bed nets so as to maximize efficiency and reduce costs, while maintaining optimal release rates.

The fact that choice studies clearly demonstrated that mosquitoes preferred entry through untreated windows than windows treated with Blends 3 and 4 is very encouraging, as it shows there is scope to develop a “push-pull” vector management strategy. Disparate “push-pull” mosquito management strategies have been evaluated with mixed results. Carbon dioxide, lactic acid, ammonia, and a range of carboxylic acids have been used to successfully simulate human odors and attract mosquitoes to baited traps such as the BG-Sentinel (Biogents, Germany) or the Centre for Disease Control (CDC)-Light trap (Menger et al. 2014, Watentena and Okoye 2019). While many of these studies clearly show the promise of artificial lures, and therefore the ”pull” elements of the systems, the spatial repellents tested were not effective enough under field or semi field conditions (Obermayr et al. 2015, Njoroge et al. 2021), especially given that even the artificial lures are unable to account for the superior attractance of a living human being in overriding the repellents (Menger et al. 2014, Njoroge et al. 2021). The combined use of an indoor repellent and outdoor baited traps reduced average nightly mosquito hut entry by 39% and 54% compared to the control-depending on *Anopheles* species (Wagman et al. 2015). Here it is proposed that the utility of the novel blends tested here and in Wood et al. (2024) could be used to supplant and improve upon the spatial repellents used in prior attempts for “push-pull” control. The development of optimally spaced and dispensed repellent blends superior to current commercial products, in conjunction with effective screens and a secondary trap baited with human odors, may result in significantly reduced biting behavior, and a concurrent increase in control efficacy.

## Conclusion

Evidence presented herein strongly demonstrates the efficacy of Blends 3 and 4 when compared to the repellent activity of several major commercial repellents. While all commercial repellents were found to exhibit some form of spatial repellency in no-choice assays, the *novo* blends were clearly superior to all of them, with Blend 3 being marginally better with smaller window coverage. In choice assays the same trend was found, with the overwhelming majority of mosquitoes choosing to fly through ports treated with commercially available repellents over the novel blends. Additionally, while Blend 4 was more effective than Blend 3, overall results demonstrates both blends were effective, suggesting that the simpler blend would be the preferable choice for further development, mirroring results found in previous developmental experiments.

## Supplementary data

Supplementary data are available at *Journal of Medical Entomology* online.

tjae137_suppl_Supplementary_Material
