# Supplementary material for: Novo plant-based mosquito repellent shows promise for exclusion of Aedes mosquitoes from “window” entry
Source: J Med Entomol. 2024 Nov 1;62(1):39–46. doi: 10.1093/jme/tjae137 (PMC11735262; doi:10.1093/jme/tjae137)
Supplement: tjae137_suppl_Supplementary_Material [file tjae137_suppl_supplementary_material.docx]

Table 1. Mean mosquito catch numbers in the presence of filter papers vs without filter papers

| Groups | Repeat no | Mean mosquito catch (%) | Min (95% confidence interval) | Max(95% confidence interval) | SD |
| --- | --- | --- | --- | --- | --- |
| 60% filter paper | 5 | 48,8 | 40,6 | 57,7 | 6,66 |
| 50% filter paper | 5 | 48,3 | 41,7 | 54,9 | 5,3 |
| 40% filter paper | 5 | 47,06 | 37,5 | 56,5 | 7,65 |
| No filter paper | 5 | 52,52 | 46,2 | 58,8 | 5,08 |

Table 2. İndependent samples t test results for the comparison of total mosquito catch with and without filter papers in the window.

| Groups | t | df | Sig (2-tailed) |
| --- | --- | --- | --- |
| 60% filter paper vs No filter paper | -0,971 | 8 | 0,360 |
| 50% filter paper vs No filter paper | -1,265 | 8 | 0,242 |
| 40% filter paper No filter paper | -1,328 | 8 | 0,221 |

Table 3. Mean mosquito catch numbers by Volunteer 1 and 2.

| Groups | Repeat no | Mean mosquito catch (%) | Min (95% confidence interval) | Max(95% confidence interval) | SD |
| --- | --- | --- | --- | --- | --- |
| Volunteer 1 | 8 | 37,8 | 30,7 | 48 | 2,41 |
| Volunteer 2 | 8 | 37,1 | 29,3 | 48 | 2,48 |

Table 4. Paired samples t-test results for the comparison of total mosquito catch between two volunteers

| Groups | t | df | Sig (2-tailed) |
| --- | --- | --- | --- |
| Volunteer 1 and Volunteer 2 | 0,164 | 7 | 0,875 |

Table 5. Mean mosquito catch numbers on the right and left side of the cage.

| Groups | Repeat no | Mean mosquito catch (%) | Min (95% confidence interval) | Max(95% confidence interval) | SD |
| --- | --- | --- | --- | --- | --- |
| Right | 8 | 36,8 | 30,7 | 48 | 2,42 |
| Left | 8 | 38,2 | 29,3 | 48 | 2,45 |

Table 6. Paired samples t-test results for the comparison of total mosquito catch between right and left side of the cage

| Groups | t | df | Sig (2-tailed) |
| --- | --- | --- | --- |
| Right and Left | -0,483 | 7 | 0,644 |

Table 7. GLMM analysis of deviance table for the no-choice olfactometric assay count model.

| Term | χ^2^ | DF | P-value |
| --- | --- | --- | --- |
| Coverage | 3.846 | 2 | 0.146 |
| ‘Control *vs*. treatment’ | 4430 | 1 | < 0.001 |
| Treatment | 23.94 | 5 | < 0.001 |
| Time | 444.2 | 1 | < 0.001 |
| Coverage: ‘control *vs*. treatment’ | 145.3 | 2 | < 0.001 |
| Concentration: treatment | 36.31 | 10 | < 0.001 |
| ‘Control *vs*. treatment’: treatment | 459.4 | 5 | < 0.001 |
| ‘Control *vs*. treatment’: time | 80.90 | 1 | < 0.001 |
| Coverage: ‘control vs. treatment’: treatment | 126.3 | 10 | < 0.001 |
